# Survival prognosis after the start of a renal replacement therapy in the Netherlands: a retrospective cohort study

**DOI:** 10.1186/1471-2369-14-258

**Published:** 2013-11-20

**Authors:** Aline C Hemke, Martin BA Heemskerk, Merel van Diepen, Willem Weimar, Friedo W Dekker, Andries J Hoitsma

**Affiliations:** 1 Organ Centre, Dutch Transplant Foundation, Leiden, the Netherlands; 2 Dutch Renal Replacement Registry, Leiden, the Netherlands; 3 Clinical Epidemiology, Leiden University Medical Centre, Leiden, the Netherlands; 4 Nephrology, Erasmus Medical Centre Rotterdam, Rotterdam, the Netherlands; 5 Nephrology, University Medical Centre Nijmegen, Nijmegen, the Netherlands

## Abstract

**Background:**

There is no single model available to predict the long term survival for patients starting renal replacement therapy (RRT). The available models either predict survival on dialysis until transplantation, survival on the transplant waiting list, or survival after transplantation. The aim of this study was to develop a model that includes dialysis survival and survival after an eventual transplantation.

**Methods:**

From the Dutch renal replacement registry, patients of 16 years of age or older were included if they started RRT between 1995 and 2005, still underwent RRT at baseline (90 days after the start of RRT) and were not registered at a non-renal organ transplant waiting list (N = 13868). A prediction model of 10-year patient survival after baseline was developed through multivariate Cox regression analysis, in one half of the research group. Age at start, sex, primary renal disease (PRD) and therapy at baseline were included as possible predictors. A sensitivity analysis has been performed to determine whether listing on the transplant waiting list should be added. The predictive performance of the model was internally validated. Calibration and discrimination were computed in the other half of the research group. Another sensitivity analysis was to assess whether the outcomes differed if the model was developed and tested in two geographical regions, which were less similar than the original development and validation group. No external validation has been performed.

**Results:**

Survival probabilities were influenced by age, sex, PRD and therapy at baseline (p < 0.001). The calibration and discrimination both showed very reasonable results for the prediction model (C-index = 0.720 and calibration slope for the prognostic index = 1.025, for the 10 year survival). Adding registration on the waiting list for renal transplantation as a predictor did not improve the discriminative power of the model and was therefore not included in the model.

**Conclusions:**

With the presented prediction model, it is possible to give a reasonably accurate estimation on the survival chances of patients who start with RRT, using a limited set of easily available data.

## Background

In the Netherlands, in recent years approximately 2000 new patients with end stage renal disease (ESRD) start chronic renal replacement therapy (RRT) every year. Even though the kidney replacement programs already exist for more than forty years, it is still not possible to predict the long term survival chances for all RRT patients during the initial phase of their therapy, using one single model.

Existing prediction models look at dialysis survival until transplantation [1], patient survival on the transplant waiting list [2-5], patient survival after transplantation [6,7], or focus on a specific patient group in which differences in treatment modality are less likely [8]. However, none of the available predication models focus on survival for the complete group of incident RRT patients, taking into account survival after dialysis combined with survival after a possible transplantation. As it is not clear at therapy initiation whether a patient will stay on dialysis, or will be listed in time and actually be transplanted, the available models cannot be used to predict survival for all patients at the start of RRT.

To be able to give a survival prognosis in an early stage of the renal replacement therapy to every patient, we need a model that predicts patient survival chances based on characteristics that are known at that point in time. In the present study, based on national data from the renal replacement registry, a prediction model on the survival prognosis for incident RRT patients in the Netherlands was developed and validated.

The objective of this study is to develop a prediction model that could be used by physicians to inform patients about their survival chances at the start of RRT, based on a few very easily obtainable variables.

## Methods

In the Dutch renal replacement registry, all ESRD patients with chronic renal replacement therapy, meaning kidney function replacement for at least 4 weeks consecutively, are registered. These patients have given written informed consent for submission of their data to the national registry. The Renine data control committee, which manages the registry, has approved the use of the data in the registry for this particular research. For this study, the baseline situation for the prognosis was the therapy at 90 days after the start of renal replacement therapy, as the intention to treat. We chose 90 days as the baseline of our study to ensure enough time to switch from a temporary needed therapy to the intended treatment and to exclude patients who only have to undergo renal replacement therapy for a short period of time. The primary renal disease (PRD) is coded in the registry according to the ERA-EDTA coding system and for our analysis grouped into 6 categories. PRD “unknown” is a specific category, as the nephrologist was not able to define the original kidney disease, so these are probably shrunken kidneys. If the PRD is missing, it could be any disease, and therefore it is different from PRD unknown. The included patients are Dutch residents of 16 years of age or older at the start of RRT, who started RRT in the period of 1995–2005, who still underwent a RRT at baseline, and who were not registered at the waiting list for another organ transplant than kidney (N = 14783). Selected patient and treatment characteristics were sex, age at start of RRT, PRD and therapy at 90 days, and the outcome was patient survival. Exclusion criteria were not registered PRD (N = 518), recovered kidney function (N = 322), lost to follow-up (N = 48), unknown kidney transplant type (N = 20), transplant failure before baseline (N = 3) or home hemodialysis as baseline therapy (N = 4). The final study group consisted of 13868 patients (Table 1). The events from 90 days after the start of RRT till death or end of the study (1/1/2010) were analyzed; the follow-up period was maximized at 10 years. For the development and validation of the prediction model, the study group was randomly divided in a development (N = 6934) and a validation group (N = 6934).

**Table 1:** Demographics of patients, N = 13868

**Patients starting a renal replacement therapy in 1995–2005, ≥16 years of age, with a registered primary renal disease and peritoneal dialysis, hemodialysis or a functioning kidney transplant at 90 days after the start**		**Total N = 13868 %**	**Development group N = 6934 %**	**Validation group N = 6934 %**	**P-value**
		%			
Sex	Male	60.2	61.1	59.4	0.04
Age group	16-44 year	17.1	17.6	16.7	0.47
	45-64 year	36.9	36.9	36.9	
	65-74 year	28.4	28.4	28.4	
	75 year or older	17.6	17.2	17.9	
Primary renal disease	Diabetes	16.7	16.6	16.8	0.71
	Renal vascular disease	25.3	25.2	25.3	
	Glomerulonephritis	12.3	12.5	12.1	
	Cystic kidney disease	9.0	8.8	9.2	
	Other diseases*	21.4	21.8	21.0	
	Unknown**	15.2	14.9	15.5	
Start year renal replacement	1995 – 2000	50.3	50.9	49.6	0.15
	2001 – 2005	49.7	49.1	50.4	
Therapy at baseline	Transplantation	3.0	2.8	3.2	0.32
	Hemodialysis	65.7	65.5	65.9	
	Peritoneal dialysis	31.3	31.7	31	

*The group “other diseases” consists of the subcategories interstitial nephritis (9.4%), other congenital and hereditary kidney diseases (1.5%), other multisystem diseases (5.4%) and other primary renal diseases (5.1%).

**The primary kidney disease “unknown” is included as a separate category in the prognosis, as these are probably shrunken kidneys, whereby it was no longer possible to determine the original disease. This is a specific recognisable category of patients, and therefore this is a separate diagnosis in the prognostic formula.

### Statistical analysis

The analysis was performed using SPSS 19 and STATA. Survival was analyzed with Kaplan Meier and log rank tests. Linearity of the influence of patient age on survival was assessed with Kaplan Meier stratified by different age groups. The proportionality assumption has been tested by visual inspection of the Schoenfeld residuals plot [9]. For the survival prognosis from 90 days after the start of RRT, multivariate Cox regression analysis was performed in the development group. The formula for the survival probability at time t, S(t), is S(t) = exp(-H(t)). Here H(t) is the cumulative hazard that is calculated from the baseline hazard (H0) as H(t) = H0(t)*exp(prognostic index). The prognostic index (PI) is the sum of the parameter estimates from the Cox regression multiplied by the patient characteristics for a specific patient. To validate the prediction model, the predictive performance was assessed by computing the calibration and the discrimination of the prediction model for the 3, 5 and 10 year survival in the validation group [10,11]. Calibration refers to the agreement between observed outcomes and predicted survival probabilities. This was measured by a) the calibration in the large, which indicates the extent that predictions are systematically too low or too high, b) a calibration plot for ten deciles according to the predicted survival, which is plotted against the observed survival, which ideally should be on the 45-degree line, and c) the calibration slope, which should be 1. The discrimination is the ability of the model to distinguish subjects with different outcomes. This was measured by the concordance (or C-) index. A C-index of 0.5 indicates the model has no discriminative power, while a model with a C-index of 1.0 has a perfect discriminative power. As a sensitivity analysis we assessed the consequences of our choice not to include information about registration on the waiting list as one of the predictors in the model and the choice for random (instead of geographical) development and validation group stratification.

## Results

The overall 10-year survival of patients on RRT at baseline was 34%. From the total cohort (N = 13868) 8418 patients died within 10 years (60.7%). The number of censored cases was 5450 (39.3%). The mean follow-up time was 5.6 years, the median was 5 years, the minimum was 0.25 and the maximum was 10 years. A prediction model of 10-year patient survival after baseline (90 days after the start of RRT) was developed through multivariate Cox regression analysis (with age, sex, PRD, and therapy at baseline as possible predictors). Based on the visual inspection of the Schoenfeld residual plots the proportionality assumption has not been rejected. Age had a linear relationship with survival. The model was developed in the development group and validated in the validation group. In the development group the number of patients at risk at 1, 3, 5 and 10 year were: 6934, 5190, 3879 and 1223. In Table 1 the development and validation group were compared and found to be not different except for a small variation in sex distribution. In Table 2 the Cox regression model is presented with the baseline hazards for the referent patient group (H0) in Table 3. To illustrate how these results can be used to compute a survival probability, consider the following example: a 50 year old male diabetic patient, that was initially treated with peritoneal dialysis has a prognostic index (PI) of ((age*0.054=)50*0.054=)2.7) + ((male=)0.067) + ((diabetes=)0.767 + ((peritoneal dialysis=)-0.131) = 3.40. Then, the 1-year survival for this patient is: exp(-(0.0030*exp(3.40))) = exp(-0.09) = 91%. The 5-year survival for this patient is: exp(-(0.0171*exp(3.40))) = exp(-0.51) = 60%. The 10-year survival for this patient is: exp(-(0.0333*exp(3.40))) = exp(-1.0) = 37%. The model can be used in a simple Excel sheet to draw an individual survival prediction curve.

**Table 2:** Cox regression model for patient mortality 90 days after start of renal replacement therapy

**Patient characteristics**		Parameter estimate*	**Hazard ratio**	**95% confidence interval**	**P-value**
Primary renal disease					<0.001
	Glomerulonephritis	*Reference*			
	Cystic kidney disease	-0.280	0.756	0.639-0.894	0.001
	Renal vascular disease	0.331	1.392	1.232-1.573	<0.001
	Diabetes	0.767	2.154	1.899-2.444	<0.001
	Other diseases	0.407	1.502	1.324-1.705	<0.001
	Unknown	0.296	1.345	1.178-1.535	<0.001
Therapy at 90 days					<0.001
	Hemodialysis	*Reference*			
	Peritoneal dialysis	-0.131	0.877	0.817-0.943	<0.001
	Kidney transplantation	-1.634	0.195	0.117-0.325	<0.001
Male sex		0.067	1.070	1.005-1.139	0.04
Age (per year)		0.054	1.055	1.052-1.058	<0.001

*The sum of (the product of) parameter estimates gives the value of the prognostic index of a patient.

**Table 3:** Baseline hazards for the referent patient group

**Period**	**Baseline hazard**
1 year	0.0030365610
2 year	0.0062357605
3 year	0.0099612016
4 year	0.0137250602
5 year	0.0171945227
6 year	0.0210066776
7 year	0.0244553143
8 year	0.0277822094
9 year	0.0310576721
10 year	0.0333077563

To assess the predictive performance of the model, the calibration and discrimination were computed in the validation group. The calibration in the large, or overall calibration, was good with a 50.4% predicted versus 49.5% observed 5-year survival and 32.9% predicted versus 34.4% observed 10-year survival.

**Figure 1:**
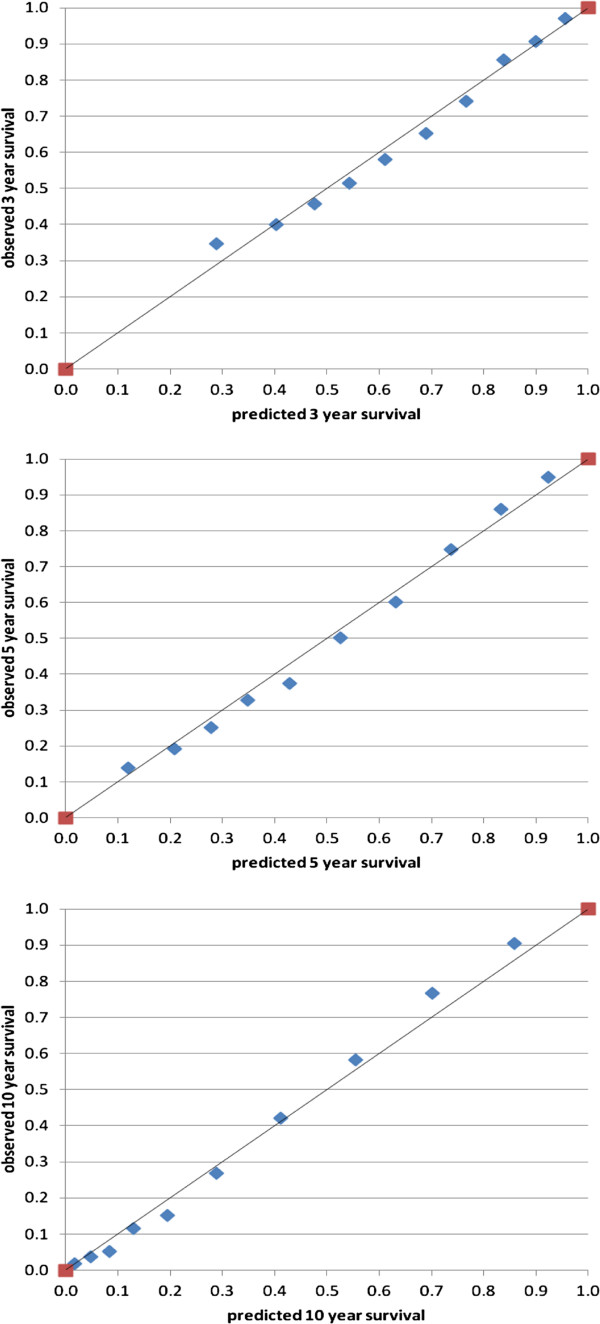
Calibration plot prediction model: observed versus predicted 3, 5 and 10 year survival.

Based on the prognostic index, ten deciles of patients were distinguished and the observed probability for 3, 5 and 10-year survival was plotted against the predicted probability in each risk stratum, constituting the calibration plot (Figure 1). The calibration slope, which ideally is 1.0, was assessed by a Cox regression analysis using the prognostic index as the only variable, and had an outcome of 0.948, 0.990 and 1.025 for the 3, 5 and 10 years survival respectively. The discriminative power of the prediction model was assessed with the concordance index and the resulting outcome of the C-index was 0.707 (95% CI: 0.698-0.717), 0.716 (95% CI: 0.708-0.724) and 0.720 (95% CI: 0.712-0.728) for the 3, 5 and 10 years survival respectively.

To show robustness of our model, we also performed sensitivity analyses.

As a first sensitivity analysis the model has been extended with the registration on the kidney waiting list at 90 days, combined with the therapy at 90 days and presented together in the model as the status at 90 days. This model had a similar validity to our current model with a discrimination of 0.724 and a calibration in the large equal, and a calibration by deciles almost equal, to that of the current model.

As the development group and validation group were very similar, we also assessed the influence of dividing the research cohort into two geographical regions, based on the ZIP-codes of the patients’ addresses. The two resulting comparable sized regions differed from each other in age-distribution, PRD-distribution, starting period, therapy at 90 days and transplantation rate. One of these regions also differed from the development group on all mentioned items; the other only differed in PRD-distribution. Two sensitivity analyses have been performed. The first was the development of a model in one region and validating the outcome in the other region. Parameter estimates of this alternative model did not differ substantially from our original (and final) model. This model had a similar validity to our final model with a discrimination of 0.711 and a calibration almost equal to that of the final model. The final model was also validated in the two separate regions. The model performed well in both regions, with a similar discrimination (C = 0.71) and calibration slopes of the prognostic index of 0.982 and 1.040 respectively (data not shown).

## Discussion

A prediction model was developed to estimate survival probabilities at 90 days based on a basic set of patient characteristics (age at start of RRT, sex and PRD) and the RRT therapy at 90 days. The main strength of the current prediction model is that it is based on the complete cohort of Dutch patients in 1995–2005. The predictive performance of the model is adequate, as demonstrated by validity tests on calibration and discrimination of this model, which could thus be used to inform patients about their survival prognosis at baseline.

The model uses treatment information at 90 days after the start of RRT, as the intention to treat. There is a clear difference in survival between patients who are on dialysis or who are being transplanted in an early stage. From previous studies we know that a better survival for kidney transplantation can be related to both advantages of the therapy as well as the better condition of the patient [2,12]. There is also a survival difference between patients starting on hemodialysis and peritoneal dialysis, which is also known from literature on this topic. Research has shown that patients starting on hemodialysis have more co-morbidities than patients starting on peritoneal dialysis [13-15]. In the prognostic formula the therapy modality therefore is included as one of the indicators of patient condition, as no other clinical information is available in the complete Dutch patient cohort. Like the ERA-EDTA, the Dutch renal replacement registry only collects a few parameters on all patients. Further research should establish whether the treatment at 90 days, in combination with age and PRD, is a good alternative for clinical parameters indicating the patient’s condition.

Registration on the kidney transplant waiting list at 90 days was not included as one of the predictors. Other studies have shown the survival benefit of patients registered at the waiting list, compared to dialysis patients not listed for transplantation [12,13,16], suggesting that this predictor is related to patient condition. For that reason we performed a sensitivity analysis to test the possible additional value of this predictor. The additional predictive performance, however, was negligible. Possibly this could indicate that there is an overlapping risk profile between dialysis patients listed and not listed for transplantation as has been shown by an American study [17], where they found that many ESRD patients viable for transplantation were not listed while higher risk patients had been listed rapidly. The comparable performance of the prediction models with and without registration on 90 days suggests that the chance to be in a better general condition and/or to be transplanted is not only reflected in registration on the waiting list, but is also covered by the other predictors (age at start, PRD and therapy at 90 days). Another reason not to include registration on the kidney transplant waiting list as a predictor in our model is the fact that the time point of registration is very arbitrary in the Netherlands. There was a very large variation in registration time in Dutch population, and many patients were registered on the waiting list for kidney transplantation after the period of 90 days, which was our baseline for inclusion. It is not very likely that there is a difference in condition between patients that are registered at 90 days or, for instance, at 91 days after the start of RRT.

Some potential limitations of this study should also be noted.

First, the moderate discriminative power of the prediction model (C-index of 0.720) shows further improvement possibilities. In this study the age, PRD, and therapy at 90 day are considered to be substitutes for more accurate clinical indicators on the condition of patients. Adding clinical patient characteristics, like GFR, proteinuria, and (historical) co-morbidities, would probably improve the individual prediction. An English study showed that the addition of comorbid condition data and laboratory data could indeed lead to improvement of the predictive power of the prognostic model from 0.69 to 0.75 [1]. On the other hand the additional effect may be limited, as age and PRD are highly correlated with comorbidity, as has also be shown by a European study [18] and a single centre study in the US [19]. It is therefore desirable to study for the Dutch situation whether clinical data correlate with data already used in this model and whether they can improve the discriminative power of the prediction model.

Another limitation of the study is that the model is only internally validated in the validation group, and the model has not been externally validated in an external cohort. It would be desirable to test the model in another patient group. This could be a patient cohort from another country or another period. The generalizability of the model to another country, however, is doubtful, as countries differ in dialysis and transplantation possibilities. This should be subject for further research. The fact that our model focuses on long term survival, makes external validation in a more recent cohort difficult. Regular evaluation of the model is needed as treatments improve in time and RRT-population, treatment possibilities and choices, both in dialysis and transplantation, change.

Finally, note that the prediction model presented in this study can only be used to inform patients about their survival chances from 90 days after their start of RRT. The patients for whom the model can be used, should have survived the first 90 days of RRT and the therapy choice for their RRT therapy has been made earlier. This prediction model is not suitable to be used for the choice of the therapy modality at the start of the RRT or for the acceptance or decline of a specific transplant kidney offer. The therapy choice should be based on preferences of the patient and physician, as is also the case for the choice to accept or decline a specific transplant kidney offer. For the choices between therapies and the probability of death on therapies new designs are currently emerging, based on competing risks instead of the Kaplan Meier method [20].

## Conclusions

In conclusion, with the presented prediction model it is possible to give a reasonably accurate estimation on the survival chances of patients who start with RRT, using a limited set of easily available data. Future research should establish whether it is possible to improve the predictive performance of the prediction model using more clinical parameters.

## Competing interests

The authors declare that they have no competing interests.

## Authors’ contributions

All authors have contributed to the results of this paper. ACH, MH and MD have worked on the design, analysis, and interpretation of the data and WW, FD en AJH have worked on the design and interpretation of the data. All authors have worked on drafting/revising the article, providing intellectual content, and final approval of the version to be published.

## Pre-publication history

The pre-publication history for this paper can be accessed here:

http://www.biomedcentral.com/1471-2369/14/258/prepub

## References

[B1] Wagner M Ansell D Kent DM Griffith JL Naimark D Wanner C Tangri N Predicting mortality in incident dialysis patients: an analysis of the United Kingdom Renal Registry Am J Kidney Dis 2011 57 894 902 10.1053/j.ajkd.2010.12.023 21489668 PMC3100445

[B2] van Walraven C Austin PC Knoll G Predicting potential survival benefit of renal transplantation in patients with chronic kidney disease CMAJ 2010 182 666 672 10.1503/cmaj.091661 20351122 PMC2855914

[B3] Rabbat CG Thorpe KE Russell JD Churchill DN Comparison of mortality risk for dialysis patients and cadaveric first renal transplant recipients in Ontario, Canada J Am Soc Nephrol 2000 11 917 922 10770970 10.1681/ASN.V115917

[B4] Medin C Elinder CG Hylander B Blom B Wilczek H Survival of patients who have been on a waiting list for renal transplantation Nephrol Dial Transplant 2000 15 701 704 10.1093/ndt/15.5.701 10809814

[B5] Schold JD Meier-Kriesche HU Which renal transplant candidates should accept marginal kidneys in exchange for a shorter waiting time on dialysis? Clin J Am Soc Nephrol 2006 1 532 538 10.2215/CJN.01130905 17699256

[B6] Jassal SV Schaubel DE Fenton SS Predicting mortality after kidney transplantation: a clinical tool Transpl Int 2005 18 1248 1257 10.1111/j.1432-2277.2005.00212.x 16221155

[B7] Kasiske BL Israni AK Snyder JJ Skeans MA Peng Y Weinhandl ED A simple tool to predict outcomes after kidney transplant Am J Kidney Dis 2010 56 947 960 10.1053/j.ajkd.2010.06.020 20801565

[B8] Couchoud C Labeeuw M Moranne O Allot V Esnault V Frimat L Stengel B A clinical score to predict 6-month prognosis in elderly patients starting dialysis for end-stage renal disease Nephrol Dial Transplant 2009 24 1553 1561 10.1093/ndt/gfn698 19096087 PMC3094349

[B9] Schoenfeld D Partial Residuals for The Proportional Hazards Regression Model Biometrika 1982 69 239 241 10.1093/biomet/69.1.239

[B10] Steyerberg EW Vickers AJ Cook NR Gerds T Gonen M Obuchowski N Pencina MJ Kattan MW Assessing the performance of prediction models: a framework for traditional and novel measures Epidemiology 2010 21 128 138 10.1097/EDE.0b013e3181c30fb2 20010215 PMC3575184

[B11] Vergouwe Y Steyerberg EW Eijkemans MJC Habbema JDF Validity of prediction models: when is a model clinically useful? Semin Urol Oncol 2002 20 96 107 10.1053/suro.2002.32521 12012295

[B12] Wolfe RA Ashby VB Milford EL Ojo AO Ettenger RE Agodoa LY Held PJ Port FK Comparison of mortality in all patients on dialysis, patients on dialysis awaiting transplantation, and recipients of a first cadaveric transplant N Engl J Med 1999 341 1725 1730 10.1056/NEJM199912023412303 10580071

[B13] Termorshuizen F Korevaar JC Dekker FW Van Manen JG Boeschoten EW Krediet RT Hemodialysis and peritoneal dialysis: comparison of adjusted mortality rates according to the duration of dialysis: analysis of The Netherlands Cooperative Study on the Adequacy of Dialysis 2 J Am Soc Nephrol 2003 14 2851 2860 10.1097/01.ASN.0000091585.45723.9E 14569095

[B14] Liem YS Wong JB Hunink MG de Charro FT Winkelmayer WC Comparison of hemodialysis and peritoneal dialysis survival in The Netherlands Kidney Int 2007 71 153 158 10.1038/sj.ki.5002014 17136031

[B15] Vonesh EF Snyder JJ Foley RN Collins AJ The differential impact of risk factors on mortality in hemodialysis and peritoneal dialysis Kidney Int 2004 66 2389 2401 10.1111/j.1523-1755.2004.66028.x 15569331

[B16] Heaf JG Lokkegaard H Madsen M Initial survival advantage of peritoneal dialysis relative to haemodialysis Nephrol Dial Transplant 2002 17 112 117 11773473 10.1093/ndt/17.1.112

[B17] Schold JD Srinivas TR Kayler LK Meier-Kriesche HU The overlapping risk profile between dialysis patients listed and not listed for renal transplantation Am J Transplant 2008 8 58 68 17979999 10.1111/j.1600-6143.2007.02020.x

[B18] van Manen JG van Dijk PC Stel VS Dekker FW Cleries M Conte F Feest T Kramar R Leivestad T Briggs JD Stengel B Jager KJ Confounding effect of comorbidity in survival studies in patients on renal replacement therapy Nephrol Dial Transplant 2007 22 187 195 16998216 10.1093/ndt/gfl502

[B19] Beddhu S Bruns FJ Saul M Seddon P Zeidel ML A simple comorbidity scale predicts clinical outcomes and costs in dialysis patients Am J Med 2000 108 609 613 10.1016/S0002-9343(00)00371-5 10856407

[B20] Beuscart JB Pagniez D Boulanger E Lessore de Sainte FC Salleron J Frimat L Duhamel A Overestimation of the probability of death on peritoneal dialysis by the Kaplan-Meier method: advantages of a competing risks approach BMC Nephrol 2012 13 31 10.1186/1471-2369-13-31 22646159 PMC3500245

